# Alveolar Pyorrhea; Its Cause, Sequelae and Cure

**Published:** 1904-09

**Authors:** D. D. Smith

**Affiliations:** Philadelphia.


### ALVEOLAR PYORRHEA; ITS CAUSE, SEQUELAE AND CURE.

By D. D. Smith, D. D. S., M. D., Philadelphia.

Read before the Philadelphia County Medical Society and the New York State Dental Society, at Albany, N. Y., May 13th, 1904.

Gentlemen: I have debated quite seriously in my own mind how best to meet present obligations and at the same time adjust this subject of pyorrhea to the needs of a constantly widening circle of earnest seekers for light and truth. My first inclinations were towards a familiar "talk" on the Cause, Sequelæ and Cure of Pyorrhea, but a somewhat critical examination of the present literature of the subject seemed to demand that a more comprehensive review should be made of some of the unsatisfactory, misleading, and positively harmful teachings which burden our text-books and magazines, and which for lack of something better have been quite generally received and adopted by the profession.

Alveolar Pyorrhea is a disease so burdened with names and vapid descriptions the wonder is that it still persists. We are indebted to the researches of Dr. Pierce for the following: "Suppuration Conjointe," "Pyorrhea Inter-alveolo-dentaire," "Gingivitis Expulsiva," "Osteo-periostiti-alveolo-dentaire," "Pyorrhea Alveolo," Cemento Periostitis," "Infectioso-alveolitis," "Calcic Inflammation," "Phagedenis Pericementitis," "Rigg's Disease," "Hematogenic Calcic Pericementitis," "Blennorrhea Alveolaris," "Gouty Pericementitis."

### PYORRHEA ALVEOLARIS IN AMERICAN TEXT-BOOK OF OPERATIVE DENTISTRY.

We may, without appreciable loss, practically disregard the articles under the above titles and many others until we come down to about 1892 to 1895, when Professor Pierce ran through the *Cosmos* a series of papers on pyorrhea, in which he aimed to make what he called the "gouty diathesis" almost wholly responsible for expression of this disorder. These articles, revised or re-written, as they appear to have been, for the *American Text-Book of Operative Dentistry,* have unfortunately done much of the thinking on this important subject for dentistry, and thus it has come to pass that Dr. Pierce's theories are widely accepted in the profession. As a consequence, it is by no means uncommon for the dentist to tell his patient that he is suffering from "gouty teeth," or "gout of the teeth;" nor for the laity to take it up and say, "My dentist says I have got gout in my teeth." Is it not time to enter an emphatic protest even to the adoption of severe criticism if necessary, against such inadequate and erroneous conceptions of this most serious mouth disorder?

Dr. Pierce defines pyorrhea as "A molecular necrosis of the retentive structures of the teeth—their ligament and pericementum—an atrophy of the alveolar walls, together with a chronic hyperemia of the gum tissue which leads to limited hypertrophy. After a variable period the teeth drop out and the morbid action ceases with their loss. An examination of the roots of the teeth before and after their exfoliation usually exhibits deposits of calculi upon their surfaces. The disease is usually, though not always, attended with flow of pus from the alveoli."

### WHAT PYORRHEA IS NOT.

Alveolar pyorrhea is not a disease transmitted through germ infection in foods, drinks, the atmosphere or by any other agency; neither is it in the remotest degree a result of some systemic disorder or constitutional vice, as rheumatism or gout. It is not necessarily "molecular necrosis of the retentive structures of the teeth;" indeed, this condition as applied to the alveoli is quite unusual. The alveolar border forms the real support for the teeth, and necrosis of it does not appear until the inflammation has engaged the gum tissue and the pericementum at some portion of the cervix with considerable violence. It may be fairly questioned whether in many cases of pyorrhea the pathological state in the alveolar border is true necrosis. It is more frequently a condition of interrupted nutrition, due to the presence of irritating inflammatory products and other accumulated toxins on and about the teeth.

WHAT REMOVAL OF EXCITING CAUSE WILL DO.

Certain it is, with the removal of the exciting cause, and properly guarding against return, the nutritive function of the alveolar border is re-established, and the retentive structures of the teeth resume their normal physiological activities. In many well attested cases new alveolar structure has been thrown out around the roots of teeth, tightening them in their sockets, and the whole recovered with normal striated gum tissue having perfect festoons.

THE LIGAMENT OF A TOOTH.

The "ligament" of a tooth should be considered as purely hypothetical. No structure analagous to a "ligament" exists in connection with a tooth when in a normal condition. Frequently when there is a gradual loosening of a molar tooth through progressive pyorrhea, there will be an exhibit of thickening of the gum tissue with increasing adhesion to one side or portion of the root of the tooth. This is but an abortive effort of nature to strengthen and retain the tooth in position. This thickening of the gum tissue—a wholly abnormal process—furnishes the only approach to a structure about the teeth which approximates a "ligament." I have never observed this tissue to be the subject of necrosis.

ONE RESULT OF PYORRHEA.

That the teeth "after a variable period" of pyorrheatic attack "drop out," and that "the morbid action ceases with their loss," is quite true.

PYORRHEA AND PUS FORMATION.

Alveolar pyorrhea is not "usually" but *always* attended with formation of pus. The very meaning of the term indicates "pus discharge," and that from about the affected teeth.

ANOTHER QUOTATION FROM "AMERICAN TEXT-BOOK."

"Clinically the cases in which these phenomena are observed may be divided into two classes: First, those in which the disease process appears to begin at the gum margin. The second class, those in connection with which there is much controversy, begin at some portion of the alveolus between the unbroken and apparently healthy gum margin and the apex of the root, the pulp of the tooth being alive."

PULP OF TOOTH ALIVE IN BEGINNING PYORRHEA.

The pulp of the tooth in the beginning of the pyorrheatic infection we believe is alive probably in every instance, for pyorrhea seldom or never originates in the tissues about a devitalized tooth or root. There may be, and often is pus discharge due to *necrosis of the root,* but this is a condition essentially different from alveolar pyorrhea.

EXAMPLES OF SIMULATED PYORRHEA.

I have observed in a few instances decay upon the proximate surfaces of lower molars occurring below old fillings, and extending to or below the gum margins, where the infection from the carious root induced simulated pyorrhea and this between two *devitalized* teeth. In these cases the removal of the irritant—the decay—and filling the cavity, wholly cured the pyorrheatic tendency. Such instances cannot be regarded as a contradiction of the statement that pyorrhea seldom or never originates in the tissues about a pulpless tooth.

TEACHINGS RESPECTING CONSTITUTIONAL PYORRHEA.

That there may be no confusion in regard to the teachings of this paper, but that they may be clearly understood, I wish to interpolate here that it is my firm conviction, having observed the clinical expressions of pyorrhea since the time it was generally known in this country as Riggs' disease, and having carefully studied it for the past ten years with the determination to ascertain its cause and to divine its meaning in the mouth, I repeat it is my firm conviction that there is no such expression of pyorrhea as that designated "Class 2d—Pyorrhea Alveolaris of Constitutional Origin"—found in the *American Text-book of Operative Dentistry,* from page 401 to 420. The cut on page 408, "Hematogenic Calcic Pericementis" (Burchard), is wholly a figment of the imagination; no pathological state resembling it ever presented in clinical experience.

THE IMPOTENCY OF DENTAL TEACHINGS.

Listen to confessions of the impotency of dentistry when it stands face to face with a grave difficulty and a diagnosis wholly wrong:

"Inasmuch as these constitutional conditions are complex in their manifestations, and their medical and hygienic management almost exclusively in the hands of the physician, the duty of the dental practitioner is confined largely to the question of diagnosis; the local treatment, however, must be varied in accordance with the peculiarities of the local pathological condition."

It seems difficult to imagine dental teachings making such lamentable confession of inefficiency.

SOME ILLUSTRATIONS IN "AMERICAN TEXT-BOOK OF OPERATIVE DENTISTRY."

I believe it is not stated that the cuts illustrating the chapter from which we have quoted in the *American Text-book of Operative Dentistry,"* were not drawn in representation of any true pathological states; nevertheless, like the one alluded to, they all seem to have been manufactured to suit conditions and descriptions *as written into the article.* Pictures of teeth, alveoli, calculi, and even of instruments, are so strained and inaccurate as to be wholly untrue to clinical observations.

THE CONSTITUTIONAL THEORY.

A recent magazine article, discussing this imaginative phase of pyorrhea, attacks its opponents with a kind of cyclonic literary *rush,* as though with one stroke of the pen to sweep from the arena all who dare oppose the theory of the constitutional origin of pyorrhea. Permit a single extract:

"The disorder to which I ask your attention is that type of chronic suppurative nechrotic inflammation in which the infection of the retentive structures of the tooth is deep-seated, where the suppurative inflammation is of the abscess type rather than the ulcerative, where salivary tartar is not the obvious irritative cause, and where local treatment alone is inadequate to effect a cure or prevent a recurrence of the disorder, and especially where some error in the nutritional process of the individual is a constant factor in the case."

As a literary composition this sentence seems faultless, but it imparts little if any get-at-able information respecting pyorrhea.

Another quotation from the same author is equally ambiguous:

"It is in connection with this condition, the hyperacid diathesis of Gauterlet, Bouchard, Michaels and others, that we find pyorrhea as a concomitant local disease."

I am unable to discern in all this, anything that can add to our knowledge of pyorrhea.

TO WHOM WE ARE INDEBTED FOR THE CONSTITUTIONAL ASPECT OF PYORRHEA.

In a "Contribution to the Study of Metabolism" it is said:

"We are greatly indebted, in my opinion, to the work and observations of Pierce, Talbott, Rhein and others, who have called our attention with much emphasis to the importance of constitutional conditions of malnutrition as predisposing factors in the causation of pyorrhea, for the more closely we investigate the matter, and the more intimately we recognize the data of nutrition in its normal and aberrant expressions, the more clearly do we see the direct bearing which nutrition exerts upon the phenomena of disease invasion."

DR. RHEIN ON PYORRHEA.

Dr. Rhein's position seems plainly stated in his article on "Studies of Pyorrhea Alveolaris," read before the 1st District Dental Society of N. Y., January 18th, 1888:

"It matters not so very much if we are correct or not as to our knowledge of the etiology. To cure the disease is the thing as far as we are concerned in our daily practice; whether the disease is purely local or only a symptom of some hidden trouble, our treatment *depends upon local remedies for its successful issue.*"

Thus it seems evident that Dr. Rhein places the emphasis *not* on the "constitutional factors," but on causes which are of a local origin. "Our treatment," he says, "depends upon local remedies for its successful issue." From the quotations made it would appear that Dr. Rhein has been misrepresented in the above article, and forced to the defence of "constitutional conditions as predisposing factors in the causation of pyorrhea," when he himself favors the belief that local agencies are the cause.

### DR. TALBOT ON PYORRHEA.

Dr. Talbot not long since, as you will recall, complained in *The Digest,* with apparent justice, that dentists are not readers. Dr. Talbot is one of the trio mentioned as emphatic defenders of the theory of a "constitutional vice in the causation of pyorrhea." So far as I have been able to discover, Dr. Talbot's contribution to alveolar pyorrhea consists in affirming that it is not pyorrhea at all, but that it is "interstitial gingivitis." I have failed to discern in just what way the nosology of this affection calls our attention "with *much emphasis* to the constitutional conditions of malnutrition as predisposing factors in the causation of pyorrhea."

The closing paragraph of "Studies in Metabolism" is as follows:

"From the view-point which I have endeavored to set before you, it *must* be obvious not only that pyorrhea, within the limitations which I have for present purposes confined that term, is the indication of a state of abnormal nutrition, but that it being a result thereof, we must seek to remove the constitutional vice before we can hope to successfully treat the local disorder."

This method of treatment is certainly unique, and reminds us of a flying-machine promoter who, to get his "flyer" into the air, proposed to suddenly move the earth away from the machine, leaving it flying in mid-air.

### "CONSTITUTIONAL VICE" CURED IN PREPARATION FOR TREATING THE "LOCAL DISORDER."

To the ordinary mind it would seem a somewhat perplexing, if not a really difficult problem, to suddenly eliminate a "constitutional vice" from the system to prepare the mouth for local treatment, especially as we, as dentists, have little or nothing to do with constitutional disorders or constitutional remedies. The article from which the above quotations are made fails to cite a single clinical case, or even to give us a hint as to what this "constitutional vice" may be; neither does it suggest a remedy or a mode of treatment.

The medical profession as yet has never presented the possibility of diagnosing general disease or of determining a constitutional vice through such chimerical methods as analyzing saliva.

### BRIEF REVIEW OF OTHER WRITERS.

Turning to other writers upon this subject, we see such frantic efforts at originality in endeavors to find causes for pyorject, we see such frantic efforts at originality in endeavors to find causes for pyorrhea, that their theories appear like some incoherent mental ataxia, and are so conspicuously absurd as to deserve only ridicule. One, in all seriousness, recently in a paper before a large dental society, ascribed a principal cause of pyorrhea to "infected toothpicks." We had supposed that wood toothpicks, or even the more expensive goosequills, to be so cheap and accessible as to preclude the use of them second hand. If they are really inoculating so many mouths with pyorrhea, it would seem to be a condition demanding the attention of boards of health. Whilst these "infected toothpicks" are given *first* place in the list of causes, the same author has another most original method of introducing his "pyogenic germ" to his destructive work, (although he says "we are unable to fix upon any specific germ"): "A deposit of calculus about a tooth proper, forcing away the soft parts, may enable the germ to enter the socket by infiltration." "Infiltration" through "calculus about a tooth *proper*," surely that would be an easy way in which to introduce this nondescript to his work of infection.

### IS THE DENTAL DEGREE "A BADGE OF PARTIAL CULTURE?"

It seems incredible that such crude pabulum, some of it evolved from a so-called analytical laboratory, can be palmed off upon the profession as a *science*, but such is the case. If we would relieve the dental degree of the stigma stamped upon it by a professor in the University of Pennsylvania, when he called it "the badge of a partial culture," and save it from overwhelming disgrace, we shall speedily relegate such infantile science as "infected tooth-picks," "infiltration of pyogenic germs," "uric acid diathesis," "gouty teeth," "constitutional pyorrhea," "gouty pericementitis" and many other absurdities, to an irretrievable oblivion.

### THE ONE MANIFESTATION OF PYORRHEA.

If we can now descend from the lofty heights of intellectual imaginations from which we have trespassed upon the unknown and unknowable, and can touch the commonalities of earth once more, I would ask your attention to a study of alveolar pyorrhea from the standpoint of its one and only manifestation:—a mouth disease of adult life due to local conditions and local causes.

Pyorrhea is a circumscribed inflammation in the tissues of the mouth; an inflammation caused wholly by the presence of natural teeth and the stagnant septic matter adherent to their surfaces.

In a beguiling pamphlet recently sent out to the dental profession by a manufacturing concern, may be found a labored attempt to present alveolar pyorrhea as a constitutional *disease* due to a so-called uric acid diathesis. This pamphlet, although signed by an M. D., is evidently from the pen of one wholly without experience. Having a meteoric surface-glow, the article is well framed to mystify and mislead respecting the true origin, and not less the treatment, of the disorder. Names, facts and inferences are speciously garbled and perverted, unmistakably in the interests of a commercial preparation recommended as a constitutional remedy.

### WHAT TEACHINGS RESPECTING CONSTITUTIONAL CAUSES HAVE DONE.

We shall do well to remember that in constitutional disturbances pyorrhea is to be reckoned with as a *cause* of disease, but never as a *result*. The etiology of pyorrhea can no longer be dismissed by the theorist with the affirmation that "it is a disease of constitutional origin;" for its manifestation near to or past middle-life only; its universal location about the roots of natural teeth; its special association with teeth of dense structure having large bell-shaped crowns practically exempt from decay; the complete immunity of all edentulous mouths, and more than all, the absolute cure without regard to constitutional conditions which attends the removal of the cause or the extraction of the teeth, all point with unerring precision to it as a trouble having positive and distinct local origin. The last analysis of the teachings which ascribe "some constitutional vice" for this condition are not only ambiguous, but they are illogical and absurd, and end in utter mental confusion. Such teachings have not only hindered, but they have practically arrested, all investigation and fixed the status of pyorrhea very generally as "incurable."

### THE ASSIGNED CAUSES FOR PYORRHEA.

The *local* cause universally assigned for alveolar pyorrhea is deposits of calculi upon the teeth. Those pretending to find it a disease ascribe it to the "gouty diathesis" or to some "constitutional *vice*." These presentations are wholly inadequate to account for the manifestations of this depraved condition.

### WHAT ACCURATE OBSERVATION REVEALS.

Accurate observations reveal the fact that deposits of calcareous matter (tartar)

upon the teeth, while causing absorption of their supporting structures, seldom or never give rise to a condition of true pyorrhea. The teeth more frequently found enveloped in masses of salivary calculi are the lower incisors and cuspids and the superior first molars. Whilst these teeth may be loosened even to the point of exfoliation through absorption of the alveolus, due to increasing deposits of tartar, this condition is not alveolar pyorrhea. The development of pyorrhea in the mouth is wholly the result of local infection; an infection arising from the breath, from vitiated mouth secretions and excretions and from chemical decomposition and decay of waste substances in the high temperature of the mouth. Toxins from these and other sources are constantly present in the mouth, and becoming cemented to the teeth chiefly by the mucous secretion, form the so-called bacterial placques, which are retained day after day, often year after year, becoming stagnant, offensive and most virulent infection.

It is common to both sexes, but rarely if ever found to originate in connection with devitalized teeth. It seldom manifests itself before middle life, and then much more commonly in mouths in which practically full and crowded dentures are found.

HEREDITARY INFLUENCE.

Heredity has no influence whatever in the development of the disorder. That heredity has an influence in determining the shape and general characteristics of the teeth, is well known and readily conceded; but while certain shapes and characteristics of the teeth are favoring conditions they are in no sense a cause of the trouble.

TEMPERAMENT A FACTOR.

Temperament as a factor in mouth pyorrhea is very imperfectly understood. As an example of wholly erroneous views respecting it, a professor speaking recently upon this point declared that the disease prevails more generally in the sanguineous temperament. The actual condition is exactly the reverse of this. It is extremely doubtful if a mouth with the typical marks of the true sanguine temperament—teeth of yellowish hue, small, rather than large, perfect in mould, shape and structure, short and having little cervical constriction, regular in development, never unduly crowded, occlusion perfect—was ever the subject of pyorrhea. I have never seen one. *Per contra,* the nervous, bilious and lymphatic temperaments, with all their compounds and admixtures, present formations and conditions of teeth and jaws which favor the retention of infection and the consequent development of pyorrhea. No classification of temperaments can possibly make the subject plain.

LOCAL CONDITIONS.

There are certain local conditions that promote the development of pyorrhea, and these demand most careful study. They are: first, the *presence of natural teeth in the mouth* (pyorrhea is impossible without natural teeth); second, the shape, character, number and placement of the teeth themselves; and third, the condition of the crown surfaces and the character and properties of the fluid environment of the teeth when the mouth is in repose. That the *presence* of natural teeth is necessary for the development of pyorrhea is manifest in the fact that it never appears in an edentulous mouth, even though the supposedly favoring constitutional factors, uric acid and gout, may be present.

The general character of the teeth, whether they are dense in structure with only a thin layer of cementum enveloping the roots—a condition necessitating limited and difficult circulation in this tissue—or whether they are softer in structure and possessed, as is usual in such cases, of a thicker cemental layer, thus permitting freer circulation in the cementum, with increased vital energy to oppose external encroachments, present important local conditions *favoring* pyorrhea in the one case and *opposing* it in the other.

The anatomical formation of the teeth also, whether having large and irregular crowns with strongly marked necks, and crowded into small, misshaped arches, or whether the reverse of these conditions prevail, viz.:—teeth small or medium in size, crowns merging into roots without marked cervical constriction, and regularly set in well-developed arches—these conditions form marked predisposing or prophylactic features in the development of pyorrhea.

The third and most important factor among the local conditions for the induction of pyorrhea is the infection inevitably present on the exposed crown surfaces of the teeth, especially the proximal surfaces. Even when considerable care is taken of them, viscid, toxic matter quickly accumulates upon untreated teeth, and is retained day after day, becoming stagnant and fetid, especially in the more inaccessible places, as between them and along the gum margins. These stagnant accumulations act to obstruct circulation in the gums, cementum and alveolus, and induce inflammation in all surrounding tissue. This infection of teeth is greatly intensified in virulence and activity by the high normal temperature of the mouth, as well as by toxic emanations from decaying tooth substance and decomposing food particles. These (and if there be any special local irritants, they are included) are the more active auxiliaries in originating pyorrheatic conditions, the subject of which are legion.

### WHERE PYORRHEA BEGINS.

The inflammation of pyorrhea begins in the tissues at some portion of the cervix of the tooth, more commonly on the lingual or palatine aspect or at some inaccessible point between the teeth, frequently between the larger molars.

In the anterior part of mouths which are the object of some care, the point of attack is usually at the angle on the right side of the lower jaw, at the interspace between the right cuspid and lateral, or it may occur one tooth farther back, between the cuspid and first bicuspid. The reason for this is found in the usually inefficient method of cleansing at this point. Using the right hand, the brush is passed over the incisors, then to the teeth upon the left side, whence it is turned to the right side of the mouth, leaving one interspace and usually one tooth untouched at the angle mentioned.

Not all teeth in any one mouth are ever the subject of pyorrhea at any one time. Strong, healthy teeth with living pulps are the special subjects of pyorrheatic attacks. Teeth which have been devitalized in comparatively young life, if the roots have been properly treated, are virtually, if not absolutely, immune. This is due to the fact that cemental structure is not solidified or changed in character or quantity after devitalization of the pulp and dentin, save as it may be increased in thickness by the pericementum through external deposit.

### EVIDENCE THAT PYORRHEA IS A LOCAL INFECTION.

I have never witnessed, in any case, the loosening and destruction of an entire set of teeth due to pyorrhea. With the displacement and loss of a single pyorrheatic tooth (much more a considerable number of such teeth) there is a marked decrease in virulence of the infection, as well as lessening of the inflammatory exudates. Other proof is in the fact that the movement of the tooth is always outward away from the point of the inflammation, as in effort to expel the irritant from the mouth. A tooth is never forced towards the center of the mouth, but always away from it, by the pyorrheatic inflammation.

Rapid elongation, protrusion or rotation of a tooth—movements at times attended with great force and considerable pain—indicate that the pericementum and cementum of the tooth are specially involved.

### THE END TO WHICH REMEDIAL EFFORT SHOULD BE DIRECTED.

To characterize the destruction of tissue in pyorrhea as "molecular necrosis" may be well enough if it is understood that the inflammation arrests, partially or entirely, the nutrition of the parts. This is especially the case in the alveolus. It is the arrest of the nutritional process which is the real cause of the excretion of the tissues at the surface. There is no zone of dead tissue exfoliated as in true necrosis. It is rather a surface necrosis. That nutrition is partially if not entirely superseded in some cases is plainly evident from the stagnant circulation, the tumefaction and purple color of the gums. The progress of the disease can only be arrested by the re-induction of normal nutrition. All remedial efforts should therefore be directed to the accomplishment of this end.

### THE TEETH A CONVENIENCE RATHER THAN A NECESSITY IN MASTICATION.

When scientifically analyzed the office work of the human teeth in the digestive process will be found far less a necessity than has hitherto been supposed. In reality they play but an inconsequential part and one easily substituted, in the preparation of foods for stomach digestion. This statement, contrary to ordinary conception and to all previous education, may seem at first an absurdity, but when seriously considered, it will be made plain that while lack of mastication by the teeth interferes greatly with our convenience and pleasure, in no true sense can mastication be classed among the *necessities* to digestion. Foods are as perfectly prepared for the human stomach and all the digestive processes without the aid of teeth as with them. This fact stands out clear and most convincingly when we consider the sick, or when we look into the mouths of the old people of today. The sick and convalescent are nourished with prepared or predigested foods, and very many of the old people have edentulous mouths, *having regained general health after the loss of all teeth;* a goodly number are using artificial substitutes which sustain about the same relation to good natural teeth that an artificial limb does to a natural one; very few have kept any considerable number of useful natural teeth to old age. In all these imperfect and inadequate conditions there is little recognition or complaint respecting inability of mastication. Good serviceable natural teeth coupled with vigorous general health in old age is an anomalous condition, while the reverse of this—few or no natural teeth and general good health—is the rule. Extensive grinding tooth-surface is not a requisite for successful mastication, especially for cooked foods; the urgent demand should be rather for the free and comfortable use of the organs of the mouth, including such teeth as may be made aseptic and serviceable. The one requisite for avoidance of chronic digestive and other complaints is a mouth with teeth perpetually void of septic infection; hence the loss of septic teeth is by no means an unmixed evil.

### PYORRHEA A CAUSE AND NOT A RESULT.

Alveolar pyorrhea with its necessary history or antecedent, long-continued septic mouth conditions, is not a result, but the unimpeachable *cause* of many serious systemic diseases, the etiology of which is not only unknown but wholly unsuspected. Its diverse local manifestations, as the displacement of the teeth, the turning of them in their sockets, the formation of pus pockets beside the roots, the loosening of the teeth and their final exfoliation, are but minor sequelæ in comparison with the constitutional manifestations seen in the slow chronic pyemic effects.

If the effects of pyorrhea were confined to its ravages in the tissues of the mouth—the wasting of the alveolus and the exfoliation of teeth—it would be of inconsiderable importance; but the pernicious emanations from twenty or more square inches of infected tooth surface, carried in inhalation directly into the circulation through the lungs, and this augmented by the perpetual ingestion of masses of mucoid toxins—pus and other pyogenic products of the disorder—develop constitutional conditions of most serious import.

### THE GRAVITY OF PYORRHEA NOT RECOGNIZED.

The gravity of states and conditions resulting from pyorrhea in the mouth has not yet been comprehended. If better understood, it would no longer be treated with comparative indifference as at present; on the contrary, it would awaken a feeling akin to alarm. As yet no comprehensive measures have been suggested by either medicine or dentistry to allay its symptoms or avert its consequences, and both professions stand in its presence today pitifully helpless.

### HOW PYORRHEA DIFFERS FROM OTHER INFLAMMATIONS.

The inflammation of alveolar pyorrhea differs from inflammations and disease conditions in other parts, in that the septic products are of necessity discharged directly into the mouth, whence they inevitably pass into the digestive tract.

Clinical observation shows the confluence of alveolar pyorrhea with rheumatism or gout to be infrequent, and if associated at all it is as cause and effect—pyorrhea the cause and rheumatism or gout the effect; a condition exactly the reverse of the "gouty diathesis" theory.

The conjunction of pyorrhea and renal complications is far more common and serious. Uremia is a usual result of pyorrhea, due in greater part to the indigestion of inflammatory products constantly disengaged in the mouth, where they are mixed with foods and drinks, and thus inevitably washed into the stomach. And here again the relation is cause and effect—pyorrhea the cause, renal complications, more commonly diabetes, the result. This has been repeatedly and indisputedly proven through cure of the pyorrhea curing the diabetic condition.

### WHERE CAUSE FIRST APPEARS.

This infection appeals for recognition first of all in septic states of the breath. Poisonous deposits upon the teeth due to toxicity of the breath are a constant menace, and principal factors in primal incitement of the disorder. Although in the aggregate such deposits may be meager in quantity, they are exceedingly virulent and violently irritative to surrounding tissues; they are rapidly augmented by alluvium from vitiated salivary and mucous secretions, and from septic excretions, and not less deleterious products from the necrotic condition of the alveolus and the perpetually irritated and inflamed gum margins at the necks of the teeth.

(Examples of breath infection appear in the surface discoloration of gold plates, gold and amalgam fillings, as well as in accumulations on the teeth.)

### A DISCOVERY DEMONSTRATED.

It is a matter for earnest felicitation that offensive breaths, a result generally of neglected mouths, repulsive mucoid oral fluids, and filthy conditions of the teeth, are wholly preventable and surely curable when they exist.

### TREATMENT.

The cause of pyorrhea being a local irritant, whether that irritant be the presence of the tooth itself or an accumulated infection on the tooth, the beginning of treatment should always be directed to the removal of the cause. Not all teeth that have been loosened through pyorrheatic infection can be redeemed and made firm, comfortable and useful, for when the disease has progressed to necrosis which involves the alveolus and pericementum, the entire circumference of the tooth, the osseous structures about the root or roots having become absorbed until the tooth is rendered hopelessly loose—alveolar tissue once absorbed is never reproduced—immediate extraction is the proper treatment; or, if through development of a pericemental abscess, as not infrequently occurs in long-standing cases of pyorrhea, especially in connection with the molars, upper or lower, there is a permanent discharge of pus from the socket, removal of the tooth is the one and only remedy.

Just here sharp distinction should be drawn between the ordinary apical alveolar abscess and the more occult and uncommon pericemental abscess. They are entirely unlike in character, in development, in prognosis and in mode of treatment. The former has been known and treated for years; the latter is of recent discovery, and is comparatively unknown. It was first differentiated by the author in 1897, and a full description of it embodied in an article which was published in the August number of the *Dental Cosmos* of that year, under the title "Pericemental Abscess." The closing paragraph of the article is as follows: "The prognosis of pericemental abscess is always unfavorable; have never been able to afford permanent nor positive relief. The only remedy—extraction of the tooth." It would be a great gain to the profession if the conditions which lead to the development of pericemental abscess were more studied and better understood.

It should be the unvarying rule in the treatment of pyorrhea to speedily rid the mouth of all teeth which cannot be made permanently comfortable and useful. Teeth, especially molars, suffering from much alveolar absorption, if they be without natural occlusive antagonism, or any that can be rotated in their sockets, even though such movement be but slight, can never be made to retighten; their retention is a source of perpetual discomfort, a hindrance to mastication, and a positive menace to the health of the mouth. In view, therefore, of the utter inutility of such teeth, any attempt to retain them is a matter of sentiment alone; every other consideration demands their immediate removal.

Having extracted such teeth as cannot be made serviceable and comfortable, effort should be directed to the removal of all infection of whatever kind from all the remaining teeth. This infection may consist of masses of calcic concretions, coatings of stagnant inspissations, accumulations of septic matter on the exposed surfaces of crowns and roots, between the teeth, in pyorrheatic pockets, or in the many concealed nooks, crannies and irregularities found in connection with the teeth.

Dentistry affecting to see pyorrhea originate in some occult and unknown constitutional cause, has wholly failed to perceive or recognize the power of septic irritants found upon the surfaces of all untreated teeth. As there is no agency more surely destructive of the teeth themselves, nor one more provocative of general systemic disturbance, than the surface infection on them, so there is no more important operation, neither a more difficult process pertaining to the teeth, than the complete and successful removal of pyorrheatic infection. Even when attempted at all, it is an operation performed with such laxity and indifference as to render it of doubtful value.

Successful treatment of pyorrhea lies in the fidelity with which the toxic irritative matter is removed from the surfaces of the teeth, and rational after-treatment maintained. Constitutional remedies are wholly without avail except as cathartics, diuretics or sudorifics they may facilitate the expulsion of poisons already absorbed. Scraping of alveolar margins, removal of vital bone, massaging of the gums, and various other impractical modes of treatment have been suggested, but none of them indicate any true appreciation of the pathological conditions involved. If necrosed bone is present, which is seldom the case, it is to be removed; beyond this, the less disturbance to the tissues involved in removal of the infection, the better the results.

All treatment should be such as surgery would institute for any wounded, abraded or excised surface in other parts, the difference being the difference in the character of the tissues with which we are dealing and in their environmental surroundings.

There is no organ or tissue in the system to which a tooth, with its anatomical and physiological complexities, can be logically compared. If relieved of infection and restored to healthful condition, being fixed in the oral cavity the teeth are at once replunged into an adverse environment, and resubjected to adverse influences.

What, then, can be done by medical interference? Practically nothing. It cannot be made too emphatic that the treatment consists in frequent, and forcible change of environment—a change from bad to good in all that pertains to the teeth, and in all mouth conditions. Every effort should be directed to re-establish healthful nutrition, which has been interrupted, if not wholly arrested, in the surrounding alveolus, pericementum and gums. No irritative toxins from the breath, from mouth fluids, offensive food particles or from other sources should be allowed on the crowns, at the necks of the teeth or in any pyorrhea pockets. Every concealed recess and nook should be reached and carefully cleansed of all accumulations with suitable instruments; and this, followed by the polishing of all exposed surfaces, including pockets, recesses and depressions, between the teeth, at the gum margins, or wherever infection may be found upon the teeth. This can be effected by hand methods only. All wheels, brushes or other revolving instruments driven by power, should be wholly discarded. Specially shaped wood points—orange-wood is to be preferred—secured in suitable holders—porte polishers—should be used for this purpose. With these all parts of the mouth and all surfaces of the teeth can be reached and operated upon. The best polishing material is a fine pumice-stone. The dry powder can be taken up and carried to place on the wood points, previously shaped and freshly dipped in water for this purpose. Free application of the pumice-stone and vigorous use of the wood points are the most efficient agents known for removing infection from the teeth and not less for placing them in the most favorable condition to avoid further deposits. This treatment should be repeated every forty-eight to seventy-two hours at the beginning, and regularly continued until there are evident indications of abatement of the inflammatory condition and the closing of the pus pockets, when the intervals of treatment may be gradually lengthened until the limit of about one month is reached.

Every mouth with pyorrheatic tendency should be rigidly subjected to this prophylaxis treatment at the hands of an expert operator for relief from the inevitable recurrence of infectious accumulations on and about the natural teeth. This treatment should be supplemented by *local* medication only. Internal remedies are not indicated except as tonics, or in special cases. Perhaps there is no better antiphlogistic or germicide for external use—and the mouth is an external cavity—than that rather disagreeable preparation known as phenol-sodique. It can be used *ad libitum* in the mouth and with great benefit, after each treatment. I have found it most efficacious applied to the gums on lint or bibulous paper.

After the second or third treatment the gums about the pyorrhea pockets and at the points between the teeth where there is great eversion of the festoons, should be touched regularly with deliquesced zinc chlorid. The handling of this preparation should always be upon wood points. A small stick with point shaped to reach the parts, may be dipped in the solution and applied as desired. It acts as a stimulant to the nutritional process. Tincture of iodin applied to the gums over a pyorrheatic cavity or tooth, is a valuable adjunct in the stimulation of healthful nutrition.

Results from the treatment as outlined have proven a source of astonishment, and not less of gratification, to many who have interestedly witnessed them. Long standing cases of pyorrhea, regarded as incurable and practically abandoned by the "constitutional cause" theorists, have readily yielded to this treatment and been cured in from six to twelve months. The recital of one case will suffice for many that might be cited in evidence:

A liberal-minded practitioner in the metropolis who had inspected some cases treated, recommended a pronounced and particularly intractable case—a gentleman aged about 55 years, with a full set of large, strong teeth, of the type peculiarly liable to pyorrheatic infection—for the treatment. The case presented no special constitutional disturbances save general malaise and an unexplainable, paroxysmal, nocturnal cough, for which he was being medicated without benefit by his family physician, who frankly admitted that he knew nothing as to the cause. Inspection of the mouth revealed general infection over all the teeth, especially the large molars and bicuspids; an offensive breath, deep pus pockets, considerable alveolar absorption and marked eversion of the gums between the molars, upper and lower. Two teeth only were decayed—the upper second molars—each having a small cavity on the distal face. The right lower second molar had a large pericemental abscess between the roots, which was discharging pus freely; this tooth was sore to pressure and quite loose. It was immediately extracted and treatment instituted for the remaining thirty-one. The records show first treatment on November eighteenth; the second on the twenty-first and the third on the twenty-sixth. There were three treatments in December, three in January, two in February, two in March and two in April. Result, after five months' treatment, cessation of all pus discharge, complete clearing of the breath, retightening of all teeth, closing of the pus pockets, reformation of festoons, perfect comfort in mastication, clearing of the skin, and entire disappearance of cough.

SERUMAL DEPOSITS.

Serumal deposits found upon the roots of teeth in pyorrheatic pockets are often difficult of diagnosis, and more difficult to remove. These deposits differ greatly in character and manner of formation from the salivary incrustations found upon the necks of teeth at the gum margins. They consist, for the most part, of smooth, hard masses of calcic matter, deposited from the stagnant fluids in the pyorrheatic pockets, cemented to the root in some irregular formation or inaccessible position, on lower teeth especially. They are not the occasion of marked inflammatory action, although the infectious matter is commonly deep seated. They create pockets beside the roots, in which deposits accumulate until the alveolus separates from the root throughout its whole extent. In many instances, the presence of these deposits can only be determined by the thickened condition of the overlying gum tissue—a characteristic of this infection—by the continued pus discharge under pressure, and by the persistent movement of the tooth, often attended with great force, in a direction opposite the accumulation. Serumal deposits destroy the life of the cementum and pericementum, and when these tissues have once separated, even though the deposits may have been perfectly removed, the vital union is never reestablished. For this reason extraction of the tooth is frequently the only remedy.

---

### GRINDING PORCELAIN TEETH FOR FULL DENTURES.

By Frederick R. Henshaw, D. D. S., Middleton, Ind.

(Written for *The American Journal of Dental Science.*)

